# Design and Fabrication of a Fast Response Resistive-Type Humidity Sensor Using Polypyrrole (Ppy) Polymer Thin Film Structures

**DOI:** 10.3390/polym13183019

**Published:** 2021-09-07

**Authors:** Mushahid Hussain, Saqib Hasnain, Nadir Ali Khan, Shehar Bano, Fazeelat Zuhra, Muhammad Ali, Munawar Khan, Naseem Abbas, Ahsan Ali

**Affiliations:** 1 Department of Electronics, University of Peshawar, Peshawar 25120, Pakistan; mushahidhussain708@gmail.com (M.H.); nakhan@uop.edu.pk (N.A.K.); munawarfata@gmail.com (M.K.); 2 Department of Mechatronics Engineering, University of Engineering and Technology, Taxila 47050, Pakistan; engrsaqibhasnain@hotmail.com; 3 School of Mechanical Engineering, Southwest Jiaotong University, Chengdu 610031, China; sherri.ali159@gmail.com; 4 Department of Chemistry, University of Peshawar, Peshawar 25120, Pakistan; fazeelat.zuhra5@gmail.com; 5 Department of Physics, University of Peshawar, Peshawar 25120, Pakistan; ali.muhammad@uop.edu.pk; 6 Sensor System Research Center, Korea Institute of Science and Technology (KIST), Seoul 136-791, Korea; 7 Department of Mechanical Engineering, Gachon University, Seongnam-Si 13120, Korea

**Keywords:** polypyrrole polymer, humidity sensor, thin film, resistive type, rapid detection

## Abstract

In this research article, an organic polymer based polypyrrole (Ppy) composite material has been synthesized and analyzed for the design and fabrication purposes of a fast-responsive, highly sensitive, and an economical resistive-type novel humidity detection sensor. This humidity sensor most suitably serves the purpose for industrial humidity (i.e., values ranging from low to high) detection applications. First, a polypyrrole composite material (a mixture of polypyrrole, polypyrrole-NiO, polypyrrole-CeO_2_, and polypyrrole-Nb_2_O_5_) has been synthesized by chemical oxidative polymerization method, and then is treated at various temperatures, i.e., 100, 150 and 200 °C, respectively. After this treatment, the synthesized samples were then characterized by using FTIR, SEM, and DTA/TGA techniques for analyzing humidity sensing properties. The polypyrrole samples with the best morphological structure and properties were then incorporated on interdigitated electrodes. For the fabrication purposes of this thin film structure, at first a few drops of polyvinyl alcohol (PVA) were placed over interdigitated electrodes (IDE) and then the synthesized polypyrrole composite was uniformly deposited in the form of a thin film over it. The plots show that this is a good resistive-type humidity detection device for the relative humidity range of 30% to 90%. The response and recovery times of this newly fabricated humidity sensor were reported to be the same as 128 s at room temperature. Additionally, the stability and the repeatability response behavior of this Ppy sensor were verified up to five cycles of multiple repetitions. This presents an excellent stability and repeatability performance of the sensor. Furthermore, the capacitances versus humidity response and recovery properties of the designed sensor were studied too. This illustrates an excellent capacitive verses humidity response and shows a linear and an active behavior. Lastly, the experimental result proves that polypyrrole composite thin film shows a reasonable best performance up to a temperature of 100 °C.

## 1. Introduction

### 1.1. Literature Review

Polypyrrole (Ppy) belongs to the family of organic polymers that can be produced by polymerization. Being a famous conductive polymer, it shows a promising behavior for the stability of a product and or device along with the conversion of the corresponding cation to anion radicals. Several methods exist for the preparation of polymers, but the most widely used methods are the electrochemical and chemically loss of electrons (i.e., oxidation). The synthesis of polymer composites from any one of these two methods depends on its type of application in practical devices. The electrochemical synthesis technique shows good results in the formation of polymers with good order. Therefore, it is the most commonly used technique to synthesize electrically conducting polymers. In 1979, Diaz et al. performed electrochemical polymerization of pyrrole, and obtained a free-standing polypyrrole film with a high conductivity of σ = 100 Scm^−1^ [1]. A general chain of polymerization scheme for polypyrrole production has been given in Figure S1a–c. After several observations, the synthesis mechanism for the polymerization process can be reduced. At the first stage, a radical cation is produced by an initial oxidation of pyrrole, which makes an electrophilic attack on a neutral molecule [2], as the electrochemical polymerization reaction only takes places when the oxidizing potential of polymer can cause the electrode surface to be zero. Furthermore, the amount of the charge absorbed during the creation of polymers is initially linearly time-dependent [3]. Finally, the complete electrochemical reaction will occur at the surface of a catalyst. Being a famous suitable conductive polymer, polypyrrole can be prepared by three known methods named as chemical vapor deposition (CVD), electrochemical polymerization, and chemical oxidation polymerization in a solution [4]. The third one is mostly used by the researchers and scientists.

Nowadays, polymeric conductive composites can be prepared by an easy and effective method which is called chemical oxidation polymerization. In the preparation of polypyrrole (H(C_4_H_2_NH)_n_H) composite, the pyrrole (C_4_H_4_NH) monomer is polymerized through the chemical oxidation polymerization method and by using oxidants such as ammonium per-sulfate ((NH_4_)_2_S_2_O_8_) within a host polymer solution. In this method, the distribution between polypyrrole and host polymer is good, and due to this, the mechanical properties and electrical conductivity of the newly prepared material considerably increases. The non-conducting state of polypyrrole is considered of having a benzenoid structure and such state is known as natural state of polypyrrole. The natural state in the benzenoid structure of polypyrrole is the same as shown in Figure S2a,b, which represents a schematic band structure of polypyrrole.

As a result of the abovementioned reaction, charge carries are performed, and monomer is polymerized due to the doping process. The formation of the structure and electronics defects as well as doping of the natural polymer take places. The first type to predominate a defect will be polarons at lower concentrations, which then give rise to two forms of two localized states within the band gap [5,6]. Latest studies related to advances in chemical synthesis of polypyrrole are presented in [7], where the authors have deposited short chain polyaniline (PANI) and Ppy based nanocomposites mixed with glucose oxidase (GOx) on a graphite rod (GR) electrode. Their results show that the designed electrodes are suitable for glucose determination in the serum samples. The complex measurements of resistivity and capacitance of an electrochemically deposited Polypyrrole are discussed in [8]. A substantial number of works on various types of synthesis methods of conducting polymers and their respective applications in the field of biosensors and biofuel cells have been conducted in [9]. More recently, a nonezymatic and an amperometric glucose biosensor was fabricated from graphite rod electrodes and then was improved with Ni-nanoparticle/polypyrrole composites, as reported in [10].

Humidity plays an important role in our daily life, such as in climate forecasting, having strong effects on our human comfort and health, in the form of moisture control in building construction and the proper running of industrial devices (electrical machines and semiconductor electronic device), etc. Humidity measurements also play a vital role in human breath monitoring system technologies [11,12,13,14] and automated manufacturing processes too [15]. Xifang et al., fabricated a humidity sensor from SPEEK/PVB composite nanofibers for breath monitoring and for non-contact sensing applications [13]. Their findings show that the addition of polyvinyl butyral (PVB) considerably improved the sensor properties such as stability, and response and recovery time. Humidity values can be acceptable up to some pre-defined ranges depending on environments of each of the abovementioned cases. However, most of the time its higher value in any environment can cause severe damages. For example, higher value of humidity around electronic devices increases conductivity of insulators; in turn this may change working properties of semiconductor devices and give rise to dangerous situations. That is why, its proper detection, measurement and control time is necessary. There exist several examples of humidity sensor applications in many sectors of our modern life and these sectors includes pharmaceutical and healthcare, automotive, domestic appliances, food and beverages, and agricultural.

Thus, different types of humidity sensors have been designed for this purpose from the applications of diverse hygroscope composite materials [16,17]. The absolute humidity and relative humidity measuring sensors are dominating the current paradigm of industrial and scientific peripheries. The design and fabrication of an effective humidity sensor is a complex task, because good performance of humidity sensors must meet many requirements including: linear curve response, higher sensitivity, physical and chemical consistency, quick response time, a low hysteresis loss, a wide operating humidity range, and lastly its affordable price. Some materials are already well known for these purposes, including polymers, ceramics, and composites, and each one of them has specific conditions for application [18,19,20,21,22,23,24,25]. For example, Rongrong et al., fabricated a capacitive-type humidity sensor from MCM-41/PEDOT composites in [22]. They have claimed that their designed sensor shows fast response, good sensing performance, and is suitable for wider humidity detection ranges.

The role of miniaturization technologies in humidity sensors offers a variety of advantages, e.g., compactness, low cost, and low density [26,27], batch fabrication [28], and low hysteresis losses [29]. It is evident from the facts that humidity plays an important role in automated industrial processes too, including the use of intelligent systems and networks. Humidity sensors are used as monitoring sensors for the diagnosis of corrosion and erosion in engineering infrastructures (e.g., civil and mechanical), and for the determination of soil moisture percentage during irrigation in agricultural projects [30]. Steps involved in advancing the sensory system of humidity includes efforts needed to increase and to improve transducer performance such as sensing elements [31,32], structure design [33,34], mechanism principle [35,36], and manufacturing and or fabrication technologies [37,38].

### 1.2. Measuring Parameters for Humidity Sensors

Humidity sensors calculate the volume of aqueous vapors present in gases. It could be a mixture of a pure gas or of an air, such as nitrogen or argon. A detailed discussion on types of humidity measuring techniques and units has been added in Supplementary Materials (see S2, Humidity Types and Units).

### 1.3. Classification of Humidity Sensors Based on Their Fabrication Materials

Relative humidity sensors are the most commonly available humidity sensors in the market. RH sensors can be sub-divided based on their operating principles. According to Yamazoe’s and Shimizu’s classification which appeared in the 1980s, various sensing elements were placed in the main groups of electrolytes, ceramics (porous), and polymers (organic) [26]. In 1995, ten years later, Traverse developed humidity sensors, mainly based on organic polymer film and porous ceramics [39]. In 2005, Chen and Lu classified the RH sensors into ceramic, semiconductors, and organic polymers. Furthermore, they had also classified absolute humidity (AH) sensors into mirror-based (hilled mirror) and slide moisture sensors [40].

All of the abovementioned kinds of humidity sensors utilize the basic principle of change in the electrical (i.e., resistance and capacitance) and physical properties of the sensitive elements, when exposed to different atmospheric humidity conditions. This provides a measure of humidity in the form of some amount of adsorption and desorption of water vapor molecules. It has been analyzed and reported that the sensitivity of humidity in porous film is higher than non-porous counterparts [41,42]. Among various other determining factors for humidity transducers, inter-granular or intra-granular porosity and pore size distribution also possess significance [43,44]. Hygrometer type humidity sensors can be made by measuring either the capacitance of sensing matters or conductance (electrical impedance), which turns into a ratio of some inorganic or organic synthetics physiology. The organic polymer film type humidity sensors are categorized into resistive, impedance [45,46] and capacitive types [47,48], and are further sub-divided into ionic conduction and electronic type sensors.

#### 1.3.1. Ceramic Humidity Sensor (Semiconductor)

Ceramic humidity sensors are metal oxide-based sensors that have advantages over polymer film type sensors from the perspective of their thermal capability, mechanical strength, resistance to chemical reactions, and physical stability. Ceramic humidity sensors are divided into impedance type sensors and the capacitive type, the impedance type sensor is then further divided into electronic conduction and ionic conduction types [49,50]. The electronic conduction and ionic conduction type sensors calculate amount of moisture through the method of measuring the variations in the conductivity of the sensing film verses various humidity levels.

#### 1.3.2. Organic/Inorganic Hybrid Composites Type Humidity Sensors

Nowadays there is an increase in the usage of different type of composite materials for humidity sensing applications; these materials are prepared by mixing several organic and inorganic substances. An important property of the ceramic system is the chemical and physical adsorption of water vapor molecules to its surface. That is why in ceramic sensors the sensitivity and response time depends upon the surface type with certain characteristic properties [39,51]. In ceramic humidity sensors the main disadvantage is that they need regular heating for the removal of dust and oil contaminants [29]. However, this problem has been resolved to some extent with the development of ionic type ceramic sensors. Ionic type ceramic sensors work at room temperature and do not require a heater [52]. In manufacturing of ceramic humidity sensors, another important thing is the proper selection of the highest temperature, to ensure the maximum interaction of water vapors and grain necks. Polymeric sensitive materials have been reported as bulk humidity sensors. This is due to variations in their dielectric permittivity or electrical conductance upon contact with water at room temperature as capacitive or resistive. Organic based polymer sensors are economical, and have simple methods of preparation, diversity, and acceptable ranges of sensitivity. However, their disadvantages includes: slow response time, short shelf lives (due to their low water durability especially at higher humidity), limitation of working in chemical and harsh environments, and weak adhesive forces with polymeric substrates. Additionally, these sensors do not work properly under firing temperatures. As seen from the issues above, it can be concluded that the most suitable solution is in the form of a hybrid primary element, and they are gaining much attention on a daily bases due to their superior features and enhanced characteristics [53].

#### 1.3.3. Polymer-Based Humidity Sensors

Macromolecules are organic polymers and represent the structure of a unit. A polymer is a combination of a carbon-hydride compound and its derivatives. The synthetic polymers are classified by monomers (i.e., small molecules). Polymers are classified by two or more various kinds of monomers which are known as co-polymers. Most of the polymeric sensors are based on porous polymer thin film (even thinner than millimeters) and having the same sensing principle as of ceramic sensors. The water vapors are coated in a polymer film with micro pores for condensation, and some measurable physical properties change due to water absorption phenomenon. The polymeric humidity sensors are categorized into two main types, the capacitive-type humidity sensor and the resistive-type humidity sensor.

##### Capacitive-Type Humidity Sensor

**Figure 1:**
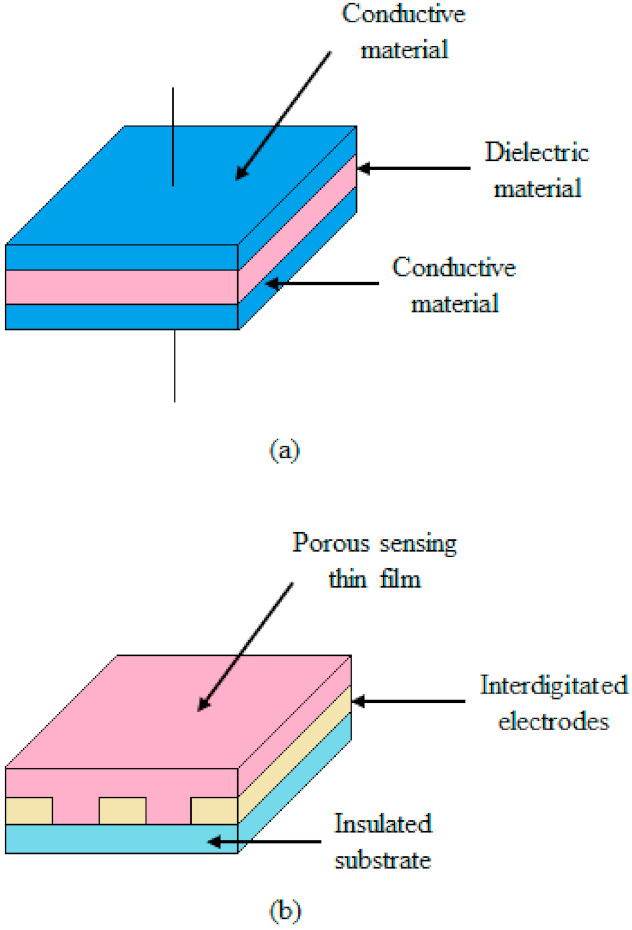
(**a**) Schematic diagram of IDE based capacitive-type thin film humidity sensor; (**b**) schematic diagram of IDE based resistive-type thin film humidity sensor.

Capacitive type humidity sensors are based on principle of change in dielectric constant in the thin film due to the absorption of water molecules. This change can be caused by the capillary condensation in porous structures or by the swelling of the polymer film. The electrode geometry and hygroscopic material determine the properties of such sensors. Because the polymer is highly sensitive to high temperatures, so nearly all the polymer-based humidity sensors operate at room temperature. Korvink et al. considered four electrode geometries for capacitive humidity sensors in [54]. The electrode geometries allow the vapors to diffuse freely into the dielectric (e.g., interdigitated electrodes (IDE)) and are favorable for faster responses. On the other hand, in thin film electrodes, the field distribution is not uniform. Nellikkal et al. presented various models of an IDE based capacitive-type and resistive-type sensor in [55]. Figure 1a shows a schematic diagram of an IDE based capacitive-type thin film single RH sensor. The IDE capacitors depend on the spacing and the area of the electrodes as shown in the Figure 1a.

##### Resistive-Type Humidity Sensors

Resistive-type humidity sensors are the sensors whose resistance changes with humidity variations. The humidity-dependent resistance changing of such sensors can be approximated by applying the following Equation (1),

(1) $$\mathrm{Log}\left(\frac{R\left(rh\right)}{Ro}\right)=\frac{loga-logr_{n}^{n}}{1+\frac{b}{r_{n}^{n}}}$$

In these sensor materials the variations in resistance are caused by a chemical reaction occurred in water molecules and surface atoms, and it could be either electronic or ionic type. A good conductive material, such as a polymer composite, produces big changes in the resistance (i.e., impedance) in the presence of water molecules, thus exhibit suitable humidity sensing properties. Figure 1b shows a schematic diagram of IDE based resistive-type thin film single RH sensor.

In this work, an organic polymer based polypyrrole (Ppy) composite material has been synthesized and analyzed for the design and fabrication purposes of a fast-responsive, highly sensitive, and an economical resistive-type novel humidity detection sensor. The novelty of this work can be seen in a number of ways, e.g., the composition of different materials used during the fabrication of this humidity sensor has not yet been reported in the entire existing literature. Furthermore, the selection of these materials has been made on the bases of their structural morphology, conductance properties, cheaper prices and easily availability. These are such attributes which can be taken care of during the fabrication of humidity sensors. Moreover, capacitive-type humidity sensors have been reported in the existing literature and are available in the market till now. However, this is a new addition in the resistive-type humidity sensors family. Properties of any sensor such as response time, recovery time, sensitivity and repeatability play an important role in the performance and suitability of the sensor. The newly fabricated sensor showed better properties in this respect too than that of existing works in this field. For example, the response and recovery times were calculated to be around 168, 1500 [56], 45, and 150 s in [57], and 180 and 60 s in [58]. However, the response and recovery times in the present work are the same and around 128 s. This in turn proves that the newly fabricated sensor in this work shows suitably enhanced sensitivity, reasonable linearity and an improved faster response and recovery time than other existing works. This work also provides a step-by-step process to fabricate a resistive-type humidity sensor from material selection to real-time response of the sensor. Apart from these mentioned new findings, most of the results presented in this work are obtained on five different frequencies, i.e., 100 Hz, 500 Hz, 1 KHz, 10 KHz, and lastly at 100 KHz; this explains the entire conductance and resistive phenomenon in a more detailed and compact way.

## 2. Material and Methods

### 2.1. Analytical Reagent Grade Pyrrole Material

Pyrrole (C_4_H_4_NH), niobium pentoxide (Nb_2_O_5_), cerium oxide (CeO_2_), nickel oxide (NiO), and anhydrous iron (III) chloride (AR-grade) were all purchased from Sigma Aldrich, Korea. Pyrrole monomers were purified under low pressure through a distillation process and then were stored in a dark place at 4 °C.

#### 2.1.1. Synthesis of Polypyrrole

For polymerization purposes of 3.4 mL pyrrole, FeC_3_ along with 25 mL of ethanol was used as a solvent with an oxidant and with anhydrous characteristics. For obtaining an ethanol solution of pyrrole, a mixture of pyrrole was first starved, and ethanol was stirred for nearly 15 min. After that, 25 mL of FeCl_3_.6H_2_O “iron (III) chloride hexahydrate” was added drop wise and with continuous stirring in this solution. The solution was left for polymerization for 24 h. The suspension was filtered out and was washed with acetone and distilled water to get ferric chloride. Finally, a black precipitate of polypyrrole was obtained. The obtained black precipitate was dried in a vacuum oven for 3 h and from 60 to 70 °C.

#### 2.1.2. Synthesis of Polypyrrole-NiO Composite

FeC_3_ was dissolved in 25 mL of ethanol and 3.4 mL of polypyrrole and stirring was performed for nearly 15 min. Then 25 mL of FeCl_4_.6H_2_O was added in a drop wise manner in an ethanol solution of pyrrole. Fine graded powder of nickel oxide (NiO) was adjusted in percentages of weights and was added to the polypyrrole solution. Then the entire mixture reaction was starved for 3 h at 0–5 °C with the help of a magnetic mixer to disperse NiO in this polymeric solution. The acquired materials were purified and properly rinsed with distilled water and acetone to eliminate the unreacted Pyrrole and the excessive amount of ferric chloride. Finally, the sample was dried in a vacuum oven for 2 h at 60 to 70 °C.

#### 2.1.3. Synthesis of Polypyrrole-Nb_2_O_5_ Composite

For this step, 3.4 mL of pyrrole was put in 25 mL ethanol and the mixture was properly allowed to dissolve, after that stirring was done on this mixture for 10 min. Then, 25 mL of FeCl_3_.6H_2_O was added in drop wise fashion in an ethanol solution of Pyrrole. Fine graded Niobium Pentoxide powder (Nb_2_O_5_) was taken in different volumes and then was added to the polypyrrole solution. Again, the sample was dried in a vacuum oven for 2 h at 60–70 °C.

#### 2.1.4. Synthesis of Polypyrrole-CeO_2_ Composite

In this step, 3.4 mL of pyrrole was added in 25 mL ethanol and then starved for almost 10 min. Then, 25 mL of FeCl_3_·6H_2_O was added drop wise in the ethanol solution of Pyrrole. Here, fine graded Cerium Oxide powder (CeO_2_) was taken in volumes of 10, 20, 30, 40, and 50, and was added to the polypyrrole solution. The mixture was starved for 3–4 h at 0–5 °C with a magnetic stirrer to disperse CeO_2_ from the polymer solution. Lastly, the sample was dried down in a vacuum oven for one hour and 30 min at 60–70 °C.

#### 2.1.5. Preparation of Pellets

**Figure 2:**
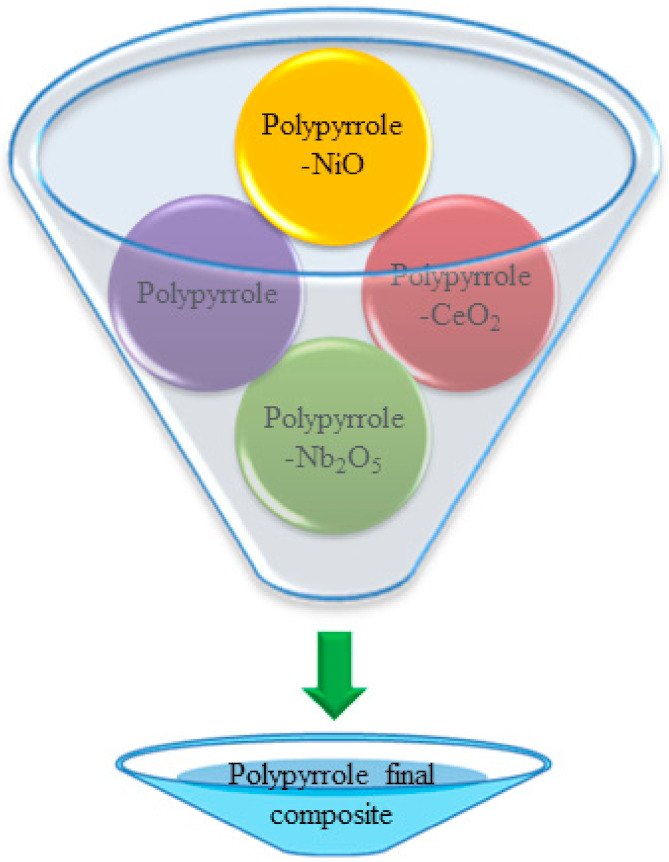
Mixing process of polypyrrole, polypyrrole-NiO, polypyrrole-CeO_2_, and polypyrrole-Nb_2_O_5_ composite materials.

In the presence of acetone medium, the powders of polypyrrole, polypyrrole-NiO, polypyrrole-CeO_2_, and polypyrrole-Nb_2_O_5_ composites obtained from above synthesis techniques were mixed (as shown in Figure 2), crushed and finely grounded in form of an agate mortar. To develop pellets of 10 mm in diameter and of a thickness of 2 mm, a pressure of 90 Mpa was applied through a hydraulic machine, and the powder was pressed. It is worth mentioning that, to obtain better contacts of sensor and temperature-dependent conductivity, the pellets of polypyrrole and its composites must be laminated with silver paste and from both sides of the surface. Additionally, a brief procedure for filtration of polypyrrole composites has been summarized in the Supplementary Materials (see S3, Procedure for Filtration of Synthesized Polypyrrole Composites).

### 2.2. Characterization Techniques

#### 2.2.1. Fourier Transform Infrared Radiation (FTIR) Spectra Analysis

A fingerprint of the newly fabricated Ppy composite molecular sample with absorption peaks has been represented by the infrared spectrum corresponding to the frequency of vibrations between bends of the atoms making up the materials. Figure S4 in the supplementary material file shows the FTIR spectra of the final Ppy composite, and this spectra has been normalized and major vibration bands are linked with different chemical groups as it can clearly be noted. FTIR spectra obtained (from paragon 1000, Perkin-Elmer, Waltham, MA, USA) for this work is with a range of wave number from 4000 to 650 cm^−1^ for duration of 64 scans, and with a 2 cm^−1^ of resolution. The numbers of materials present in Ppy composite are directly indicated by the size of peaks in the spectrum.

#### 2.2.2. Differential Thermal Analysis (DTA)/Thermo-Gravimetric Analysis (TGA)

**Figure 3:**
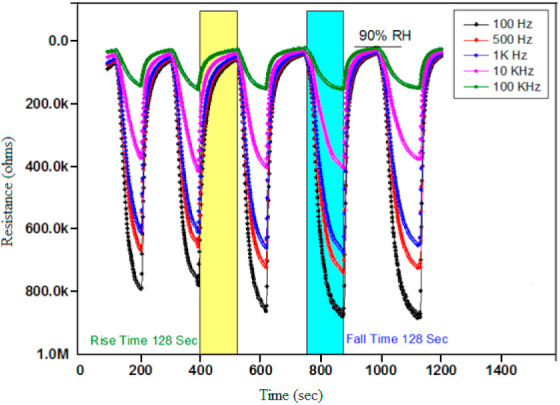
DTA/TGA results of humidity sensor of PVA polypyrrole composite thin film structure at five different frequencies and at changing RH from 30% to 90%.

Thermo-Gravimetric Analysis (TGA) is the study of changes in mass of samples occurring in variations of heat [59]. The Ppy composite based thin film sensor was placed in a sealed and controlled humidity glass chamber for absorption and desorption analysis purposes. A newly prepared Ppy composite thin film structure has been characterized on the basis of these techniques as shown in Figure 3. Figure 3 is showing thermal absorption and desorption properties of this Ppy composite at five different frequencies with respect to changing RH from 30% to 90%. It can be concluded that the absorbed water molecules readily leave the outer thin film sensing surface of the Ppy composite material in the recovery process, whereas active unoccupied places of the surface become available for the adsorption of freshwater molecules in the succeeding sensing cycles. As a result, this produces a thoroughly unchanged rise and fall response, as shown in the repeated detection process. More briefed details related to Figure 3 can be found in Section 3.1.

#### 2.2.3. Scanning Electron Microscopy (SEM)

**Figure 4:**
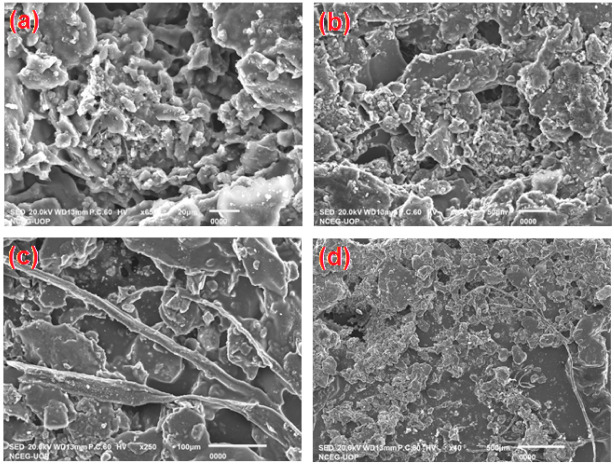
SEM microstructures of Ppy composite sample; (**a**) sample image at 20 µm, (**b**) sample image at 50 µm, (**c**) sample image at 100 µm, (**d**) sample image at 500 µm showing larger empty spaces.

SEM was developed for scanning the surface of a sample of a substance. SEM can be used to examine and obtain certain information about chemical compositions and surface topography that compose the crystalline structure, giving a magnified image of the sample of a substance [60]. The special microstructures and structural morphology of the Ppy composite material was carefully conducted by using SEM (from JSM-IT100, Japan) as shown in Figure 4. Figure 4 shows that the Ppy composite material is intergranular porous, agglomerated and gives a sheet-like structure. It was found from SEM analysis that there a larger number of empty spaces (Figure 4d) exists in the microstructure of the Ppy composite powder, which will provide a larger porous surface area for water molecules absorption and the desorption process. This will notably enhance the resistance changes of this material. In addition, these kinds of microstructural characteristics will play an important role in enhancing the Ppy composite based thin film sensor performance parameters such as rise time, fall time, and sensitivity response behavior.

### 2.3. Fabrication of the Humidity Sensor of PVA Polypyrrole

**Figure 5:**
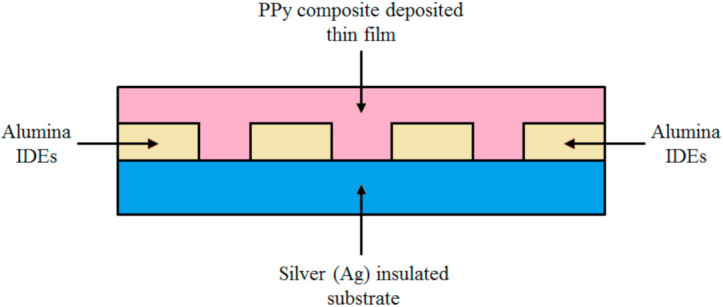
Cross-sectional view of the newly fabricated Ppy composite thin film resistive-type humidity sensor.

**Figure 6:**
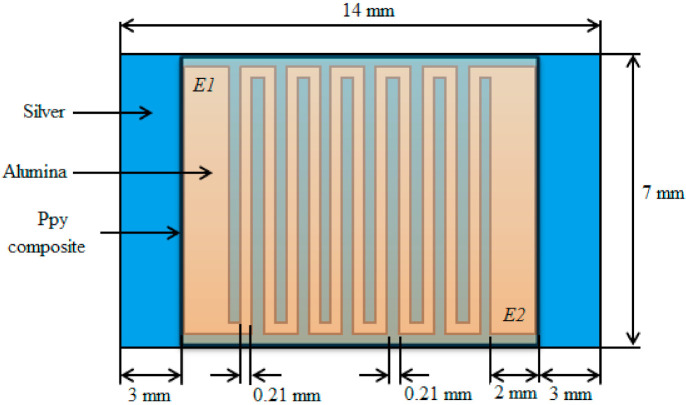
Actual schematic configuration of interdigitated electrodes in the newly fabricated thin film sensor for measuring humidity (top view).

For fabrication purposes of the Ppy composite based thin film sensor, at first a few drops of polyvinyl alcohol (PVA) were placed over an interdigitated electrodes (IDE) and then the synthesized polypyrrole composite was uniformly deposited in form of a thin film over it. Note that the composition of PVA and Ppy is different: the amount of Ppy is five times the amount of PVA. Figure 5 depicts a simplified cross-sectional view of the Ppy composite based thin film fabricated resistive-type humidity sensor. Where, the bottom layer is of silver (Ag), the IDEs are made up of alumina (Al_2_O_3_), and the top deposited layer is of Ppy composite material. Figure 6 shows the actual schematic diagram of an interdigitated electrode configuration for sensor application in more details. Where, E1 and E2 are the two electrodes to connect this thin film structure with the interfacing module.

## 3. Results and Discussion

### 3.1. Stability and Repeatability

Figure 3 shows the response (rise) and recovery (fall) times of this newly fabricated Ppy humidity sensor, which both are the same as 128 s at room temperature when fluctuating between humidification and dehumidification values of 30% and of 90% RH occurred, respectively. The performance and efficiency of the sensor as a resistance function at different frequencies (e.g., ranging from 100 Hz to 100 KHz) are measured and are plotted too. The stability and the repeatability of this Ppy composite sensor has been verified up to five cycles of multiple repetitions and at different frequencies of 100 Hz, 500 Hz, 1 KHz, 10 KHz, and lastly at 100 KHz. This presents that the newly fabricated Ppy based resistive-type thin film sensor is exhibiting an excellent stability and repeatability performance behavior for repeated cycle of experiments. Figure 3 also shows that the Ppy based composite thin film sensor values are more responsive and sensitive at lower frequencies such as 100 and 500 Hz, and are less responsive and insensitive at higher frequencies of 10 and 100 KHz.

### 3.2. Capacitance verses Humidity

**Figure 7:**
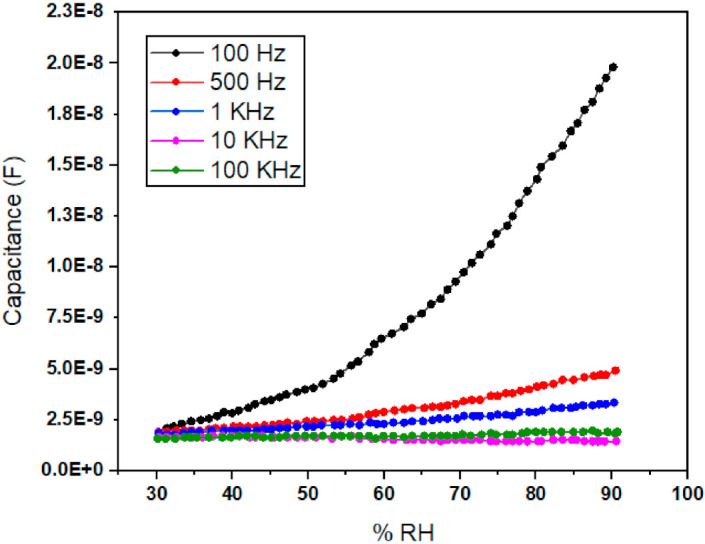
Capacitive verses % RH response of Ppy composite based thin film sensor at five different frequencies and 1 volt.

The plot comprising the capacitance verses % RH of the newly fabricated device was also obtained and is shown in Figure 7. Figure 7 shows notable variations in the capacitance with increasing % RH levels at five different operating frequencies (e.g., Freq 1 = 100 Hz, Freq 2 = 500 Hz, Freq 3 = 1 KHz, Freq 4 = 10 KHz, and Freq 5 = 100 KHz) at constant electrical potential of 1 volt. This can be confirmed that our plotted response behavior is in accordance with the previous research results as explained in [61]. It is obvious from Figure 7 that the changes in capacitance values are directly dependent on the operating frequencies. Additionally, it can be noted from the Figure 7 that the capacitance values change linearly with a change in % RH at a lower frequency of 100 Hz, starting from 60% RH. Capacitance values become constant for higher operating frequencies, such as 10 and 100 KHz.

It is further revealed from the Figure 7 that the sensor values remain more responsive and sensitive at lower frequencies and less responsive and insensitive at higher frequencies of 100 and 100 KHz, respectively. Less responsive and insensitive behaviors can be associated with the weak polarization of Ppy composite thin film molecules. Furthermore, highest values of sensitivity for this thin film Ppy composite sensor could be obtained at 1 V (Vrms) and frequencies equal to or lower than 100 Hz. However, low operating frequencies are associated with some problems such as humidity sensor stability and repeatability. That is why, humidity sensors should not be operated at lower frequencies to avoid bad response. So there exists a tradeoff between these sensor’s performance parameters. Figure 7 also proves that when % RH values increase, the water molecules adsorbed by the Ppy composite thin film also increases, and subsequently capacitance of the sensor also increases.

**Figure 8:**
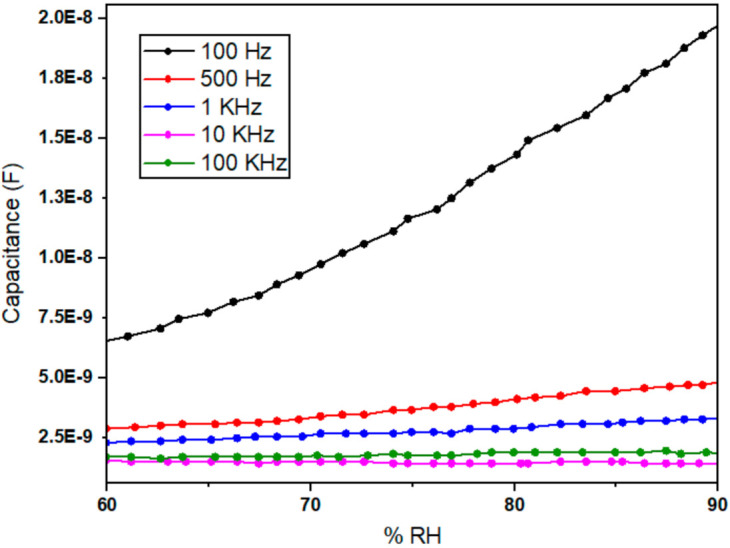
Magnified capacitive verses % RH response of Ppy composite based thin film sensor from 60% to 90% humidity range and showing linear behavior for a lower frequency of 100 Hz.

Figure 8 shows the magnified capacitive verses % RH response of the Figure 7. Figure 8 shows that the above discussed linear behavior is more obvious from 60% to 90% RH of humidity values and at the lowest operating frequency of 100 Hz. This illustrate that the newly fabricated Ppy composite thin film sensor response is more active and accurate in this range. This also confirms that the changes in the capacitance values are more significant at lower frequencies (i.e., lower than or equal to 100 Hz) than at higher frequencies (i.e., higher than 100 Hz).

### 3.3. Resistance verses Humidity

**Figure 9:**
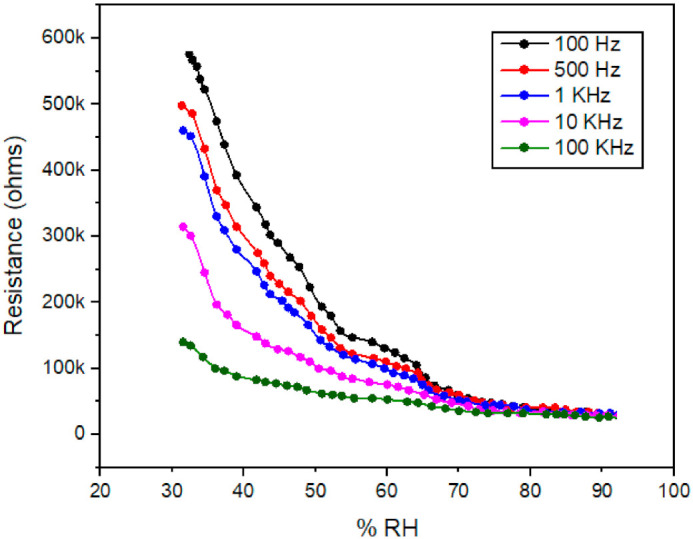
Resistance verses % RH response of Ppy composite based thin film sensor at five different frequencies and 1 volt.

Subsequently, the newly fabricated device was placed in the humidity test chamber again and the resistance verses % RH values of the device were obtained for the same operating frequencies (i.e., Freq 1 = 100 Hz, Freq 2 = 500 Hz, Freq 3 = 1 KHz, Freq 4 = 10 KHz, and Freq 5 = 100 KHz) and at a constant 1 volt. Figure 9 shows the variations in the electrical resistivity as a function of % RH of this resistive-type thin film structure. In general, it is noted that when the % RH increases, the sensor resistivity decreases for all values of the operating frequencies, which in turns increases conductivity of the sensor. It can be predicted that when % RH increases, it provides larger amount of energy to electrons present in the valence band of the sensor material. These higher energy electrons jump to the conduction band and results an increase in the conductivity and decrease in the resistivity. It could be assumed that the thermal curling process will affects the Ppy composite chain alignment, which can decrease the resistivity of this thin film structure.

It can be carefully observed that when the value of % RH rises then the value of resistance reduces in an exponential form (as discussed in [61]) for lower values of operating frequencies, such as at 100 Hz, and that an inverse relationship exists between these two parameters. Additionally, the response behavior looks slower and less sensitive for higher values of frequencies, such as at 100 KHz.

### 3.4. Hysteresis of Ppy Thin Film Sensor

**Figure 10:**
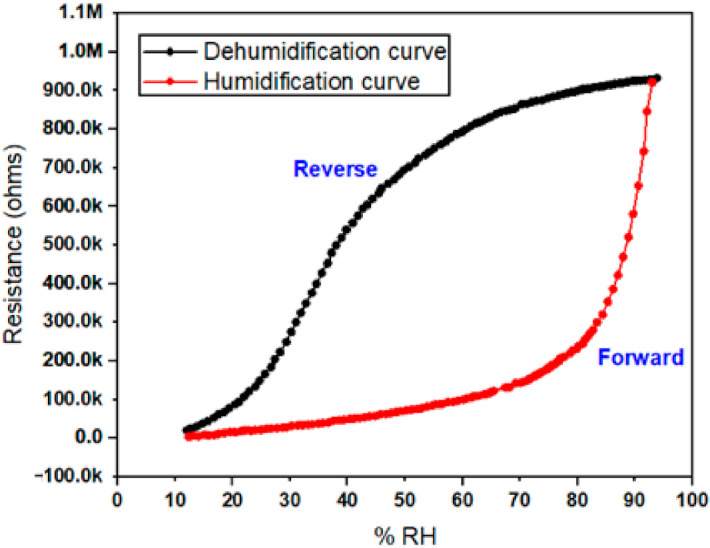
Resistance verses % RH graph showing humidification and dehumidification hysteresis cyclic curve at a lower frequency of 100 Hz and at 1 volt.

A hysteresis plot is another important feature among several others of humidity detection sensors which highlights their performance and various other properties. It shows a lag between humidification and dehumidification of the Ppy composite thin film sensor in Figure 10. To observe the hysteresis behavior of the sensor’s humidification and dehumidification, cycle curves in form of a resistance verses time plot on 100 Hz of maximum operating frequency has been drawn. It can be observed that the humidity level was first increased from 12 to 90% RH in smaller steps of % RH and then was decreased down to 12% RH again.

This plot shows that during the humidification curve (i.e., forward curve) the value of resistance increases with an increase in time. In the same way, during the dehumidification curve (i.e., reverse curve) the value of resistance decreases with a decrease in time. In such types of ideal cases, it can be stated that the forward process and reverse process are almost identical to each other with a minimum hysteresis loss value, this in turn also demonstrates that the stability performance of the sensor is reliable and can be used for application purposes.

### 3.5. Real-Time Humidity Response of the Sensor

Real-time visualization of humidity values of the sensor plays an important role in the final verification process. Figure S5 is showing the real-time humidity response behavior of this Ppy composite based thin film sensor for a time period of two days as measured on 26 December 2020 and 27 December 2020. The newly fabricated sensor was thoroughly tested for various temperature ranges, and it showed a promising response even at higher temperatures too, i.e., 100 °C.

### 3.6. Equivalent Circuit Diagrams of Ppy Composite Sensor

**Figure 11:**
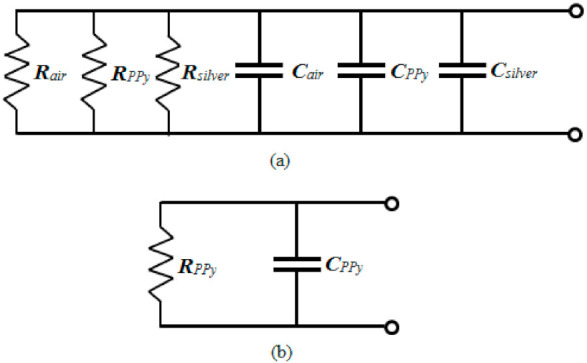
(**a**) Detailed schematic diagram of electrical equivalent circuit of Ppy composite thin film sensor and (**b**) simplified schematic diagram of electrical equivalent circuit of Ppy composite thin film sensor.

Schematic diagrams of Ppy composite polymer resistive-type thin film sensors (of alumina as IDEs and Ppy as deposited surface) in a detailed and equivalent form are drawn in Figure 11a,b, respectively. In Figure 11a, C*_air_* and R*_air_* denote the capacitance and resistance due to the dielectric of air occupied in the pore area. The C*_PPy_* and R*_PPy_* give the capacitance and resistance due to the dielectric of Ppy composite itself when exposed to the surrounding environmental parameters such as relative humidity, light, and temperature, respectively. The capacitance, C*_silver_*, and resistance, R*_silver_*, are due to the dielectric of the silver substrate. As, the electrical properties of Ppy thin film are dominant over other materials properties, that is why the capacitance and resistance properties of the PPy are only taken into account, and an equivalent circuit has been drawn in Figure 11b.

## 4. Conclusions and Future Recommendations

An organic polymer based polypyrrole composite material (a mixture of polypyrrole, polypyrrole-NiO, polypyrrole-CeO_2_, and polypyrrole-Nb_2_O_5_) has been synthesized and analyzed for the design and fabrication purposes of a novel resistive-type humidity detection sensor. The polypyrrole samples with best morphological structure and properties were incorporated on alumina interdigitated electrodes. The synthesized polypyrrole composite has been treated at various temperatures, i.e., 100, 150, and 200 °C, respectively. It was observed from the resistance verses % RH plot that if the % RH rise’s then the resistance reduces exponentially. Additionally, a linear behavior was found between % RH and capacitance values for lower range of operating frequencies. The FTIR spectrum of Ppy composite is predicting the presence of hydroxyl (OH) by showing a very strong and broad peak at 3299 cm^−1^.

TGA/TDA analysis was also applied to estimate the thermal stability of Ppy composite, and it was noted that the thermal stability of Ppy composite attained at approximately 250 °C. It was further found at a temperature of 100 °C, that FTIR analysis of Ppy composite shows that all peaks of Ppy were present in the FTIR spectra except for the water molecules. Furthermore, the experimental result proves that the polypyrrole composite shows a reasonable best efficiency and performance at a temperature of 100 °C. So, this sensor is best suited for such temperature environment applications.

The plots show that this is a good resistive-type humidity detection device in the relative humidity range of 30% to 90%. The response and recovery times of this newly fabricated humidity sensor were measured to be the same as 128 s at room temperature when fluctuating between humidification of 30% and dehumidification of 90% in RH occurred. Additionally, the stability and the repeatability of this Ppy sensor were verified up to 5 cycles of multiple repetitions. It is indicated that this sensor is capable of measuring humidity levels above 30% and below 90% and with full scale recovery. This presents an excellent stability and repeatability performance of the sensor.

Ppy and its composites-based sensors have various potential applications, such as in tissue engineering, instrumentation, agriculture, automated industrial manufacturing processes and energy storage devices, etc. Ppy can also be used as a polyelectrolyte-based type resistive sensor because of its hydrophilic nature. By applying further optimization techniques, a continuous, homogenous, and a highly porous Ppy can be obtained which has numerous other benefits. Additionally, doping of Ppy and by adjusting the efficiency (e.g., recovery and response time) of the synthesized Ppy, performance of IDE sensors can considerably be improved.

## Data Availability

Data is contained within the article.

## Supplementary Materials

The following are available online at https://www.mdpi.com/article/10.3390/polym13183019/s1, Figure S1: Polypyrrole polymerization scheme (a): Generation of free cation radical, (b): polymer chain propagation, (c): formation of a polymer chain, Figure S2: Types of bonds in natural state in the benzenoid structure of Polypyrrole (a): aromatic, (b): quinonoid structure, Figure S3: Correlation among dew/frost point, PPMv, and % RH, Figure S4: FTIR spectra of Ppy composite, Figure S5: Real-time humidity values of the sensor.

## References

[1] Diaz A.F., Kanazawa K.K., Gardini G.P. (1979). Electrochemical polymerization of pyrrole. J. Chem. Soc. Chem. Commun..

[2] Cheng G., Ding J., Zhang Z., Ling Z., Pu H. (2012). Study on the preparation and multiproperties of the polypyrrole films doped with different ions. Surf. Interface Anal..

[3] Harraz F.A., Salim M.S., Sakka T., Ogata Y.H. (2008). Hybrid nanostructure of polypyrrole and porous silicon prepared by galvanostatic technique. Electrochim. Acta.

[4] Yoshino K., Hayashi S., Sugimoto R.-I. (1984). Preparation and properties of conducting heterocyclic polymer films by chemical method. Jpn. J. Appl. Phys..

[5] Nguyen T.P., Rendu P.L., Tran V.H., Parkhutik V., Fenollosa Esteve R. (2000). Electrical and optical properties of conducting polymer/porous silicon structures. J. Porous Mater..

[6] Harraz F.A. (2006). Electrochemical polymerization of pyrrole into nanostructured p-type porous silicon. J. Electrochem. Soc..

[7] German N., Ramanaviciene A., Ramanavicius A. (2021). Dispersed conducting polymer nanocomposites with glucose oxidase and gold nanoparticles for the design of enzymatic glucose biosensors. Polymers.

[8] Samukaite-Bubniene U., Valiūnienė A., Bucinskas V., Genys P., Ratautaite V., Ramanaviciene A., Aksun E., Tereshchenko A., Zeybek B., Ramanavicius A. (2021). Towards supercapacitors: Cyclic voltammetry and fast fourier transform electrochemical impedance spectroscopy based evaluation of polypyrrole electrochemically deposited on the pencil graphite electrode. Colloids Surf. A Physicochem. Eng. Asp..

[9] Ramanavicius S., Ramanavicius A. (2021). Charge transfer and biocompatibility aspects in conducting polymer-based enzymatic biosensors and biofuel cells. Nanomaterials.

[10] Emir G., Dilgin Y., Ramanaviciene A., Ramanavicius A. (2021). Amperometric nonenzymatic glucose biosensor based on graphite rod electrode modified by Ni-nanoparticle/polypyrrole composite. Microchem. J..

[11] Xiaoqiang L., Qian S., Yan K., Yanan Z., Zengyuan P., Yang J., Mengjuan L., Chronakis I.S. (2020). Self-powered humidity sensor based on polypyrrole modified melamine aerogel. Mater. Lett..

[12] Mazhar S., Qarni A.A., Haq Y.Q., Haq Z.U., Murtaza I., Ahmad N., Jabeen N., Amin S. (2020). Electrospun PVA/TiC nanofibers for high performance capacitive humidity sensing. Microchem. J..

[13] Li X., Zhuang Z., Qi D., Zhao C. (2020). High sensitive and fast response humidity sensor based on polymer composite nanofibers for breath monitoring and non-contact sensing. Sens. Actuators B Chem..

[14] Dai J., Zhao H., Lin X., Liu S., Fei T., Zhang T. (2020). Design strategy for ultrafast-response humidity sensors based on gel polymer electrolytes and application for detecting respiration. Sens. Actuators B Chem..

[15] Kastner W., Neugschwandtner G., Soucek S., Newman H.M. (2005). Communication systems for building automation and control. Proc. IEEE.

[16] Wise D.L. (1998). Electrical and Optical Polymer Systems: Fundamentals: Methods, and Applications.

[17] Dian J., Konečný M., Broncová G., Kronďák M., Matolínová I. (2013). Electrochemical fabrication and characterization of porous silicon/polypyrrole composites and chemical sensing of organic vapors. Int. J. Electrochem. Sci..

[18] Funt L., Tanner J., Weinger N.L. (1975). Electrochem, Synth, Poly, Tech, Electro-Org, Synth.

[19] Pokhrel S., Jeyaraj B., Nagaraja K.S. (2003). Humidity-sensing properties of ZnCr_2_O_4_–ZnO composites. Mater. Lett..

[20] Chen Y.S., Li Y., Yang M.J. (2005). Humidity sensitive properties of NaPSS/MWNTs nanocomposites. J. Mater. Sci..

[21] Arrizabalaga O., Velasco J., Zubia J., De Ocáriz I.S., Villatoro J. (2019). Miniature interferometric humidity sensor based on an off-center polymer cap onto optical fiber facet. Sens. Actuators B Chem..

[22] Qi R., Zhang T., Guan X., Dai J., Liu S., Zhao H., Fei T. (2020). Capacitive humidity sensors based on mesoporous silica and poly(3,4 ethylenedioxythiophene) composites. J. Colloid Interface Sci..

[23] Karunarathne T.S.E.F., Wijesinghe W.P.S.L., Rathuwadu N.P.W., Karalasingam A., Manoharan N., Sameera S.A.L., Sandaruwan C., Amaratunga G.A., De Silva S.G.M. (2020). Fabrication and characterization of partially conjugated poly (vinyl alcohol) based resistive humidity sensor. Sens. Actuator A Phys..

[24] Park Y.-J., Lee S., Kim B., Kim J.-H., So J.-H., Koo H.-J. (2020). Impedance study on humidity dependent conductivity of polymer composites with conductive nanofillers. Compos. B Eng..

[25] Lang C., Liu Y., Cao K., Li Y., Qu S. (2019). Ultra-compact, fast-responsive and highly-sensitive humidity sensor based on a polymer micro-rod on the end-face of fiber core. Sens. Actuators B Chem..

[26] Yamazoe N., Shimizu Y. (1986). Humidity sensors: Principles and applications. Sens. Actuators.

[27] Fagan J.G., Amarakoon R.W. (1993). Reliability and reproducibility of ceramic sensors. III: Humidity sensors. Am. Ceram. Soc. Bull..

[28] Goldberg H.D., Brown R.B., Liu D.P., Meyerhoff M.E. (1994). Screen printing: A technology for the batch fabrication of integrated chemical-sensor arrays. Sens. Actuators B Chem..

[29] Kulwicki B.M. (1991). Humidity sensors. J. Am. Ceram..

[30] Dean R.N., Rane A.K., Baginski M.E., Richard J., Hartzog Z., Elton D.J. (2011). A capacitive fringing field sensor design for moisture measurement based on printed circuit board technology. IEEE Trans. Instrum. Meas..

[31] Lin W.D., Chang H.M., Wu R.J. (2013). Applied novel sensing material graphene/polypyrrole for humidity sensor. Sens. Actuators B Chem..

[32] Xu C.N., Miyazaki K., Watanabe T. (1998). Humidity sensors using manganese oxides. Sens. Actuators B Chem..

[33] Pelino M., Colella C., Cantalini C., Faccio M., Ferri G., D’Amico A. (1992). Microstructure and electrical properties of an α-hematite ceramic humidity sensor. Sens. Actuators B Chem..

[34] Klym H., Hadzaman I., Shpotyuk O., Brunner M. P3.6-multifunctional T/RH-sensitive thick-film structures for environmental sensors. Proceedings of the SENSOR+TEST Conferences 2011 Proceedings on SENSOR.

[35] Traversa E., Bearzotti A. (1995). A novel humidity-detection mechanism for ZnO dense pellets. Sens. Actuators B Chem..

[36] Gusmano G., Montesperelli G., Nunziante P., Traversa E. (1993). Study of the conduction mechanism of MgAl_2_O_4_ at different environmental humidities. Electrochim. Acta.

[37] Smetana W., Unger M. (2008). Design and characterization of a humidity sensor realized in LTCC-technology. Microsyst. Technol..

[38] Karimov K.S., Cheong K.Y., Saleem M., Murtaza I., Farooq M., Noor A.F.M. (2010). Ag/PEPC/NiPc/ZnO/Ag thin film capacitive and resistive humidity sensors. J. Semicond..

[39] Traversa E. (1995). Ceramic sensors for humidity detection: The state-of-the-art and future developments. Sens. Actuators B Chem..

[40] Chen Z., Lu C. (2005). Humidity sensors: A review of materials and mechanisms. Sens. Lett..

[41] Connolly E.J., O’Halloran G.M., Pham H.T.M., Sarro P.M., French P.J. (2002). Comparison of porous silicon, porous polysilicon and porous silicon carbide as materials for humidity sensing applications. Sens. Actuator A Phys..

[42] Salehi A., Kalantari D.J., Goshtasbi A. Rapid response of Au/porous-GaAs humidity sensor at room temperature. Proceedings of the 2006 IEEE Conference on Optoelectronic and Microelectronic Materials and Devices.

[43] Yang B., Aksak B., Lin Q., Sitti M. (2006). Compliant and low-cost humidity nanosensors using nanoporous polymer membranes. Sens. Actuators B Chem..

[44] Shah J., Kotnala R.K., Singh B., Kishan H. (2007). Microstructure-dependent humidity sensitivity of porous MgFe_2_O_4_–CeO_2_ ceramic. Sens. Actuators B Chem..

[45] Santha H., Packirisamy M., Stiharu I., Li X., Rinaldi G. (2005). A polyimide based resistive humidity sensor. Sens. Rev..

[46] Cho N.B., Lim T.H., Jeon Y.M., Gong M.S. (2008). Inkjet printing of polymeric resistance humidity sensor using UV-curable electrolyte inks. Macromol. Res..

[47] Dwiputra M.A., Fadhila F., Imawan C., Fauzia V. (2020). The enhanced performance of capacitive-type humidity sensors based on ZnO nanorods/WS_2_ nanosheets heterostructure. Sens. Actuators B Chem..

[48] Jachowicz R.S., Senturia S.D. (1981). A thin-film capacitance humidity sensor. Sens. Actuators.

[49] Pal B.N., Chakravorty D. (2006). Humidity sensing by composites of glass ceramics containing silver nanoparticles and their conduction mechanism. Sens. Actuators B Chem..

[50] Jeseentharani V., Reginamary L., Jeyaraj B., Dayalan A., Nagaraja K.S. (2012). Nanocrystalline spinel Ni_*x*_Cu_0.8-*x*_Zn_0.2_Fe_2_O_4_: A novel material for humidity sensing. J. Mater. Sci..

[51] Krutovertsev S.A., Tarasova A.E., Krutovertseva L.S., Chuprin M.V., Ivanova O.M., Sazhinev Y.S. Technology and characteristics of microhumidity sensors. Proceedings of the 2011 IEEE 16th International Solid-State Sensors, Actuators and Microsystems Conference.

[52] Jain M.K., Bhatnagar M.C., Sharma G.L. (1999). Effect of Li^+^ doping on ZrO_2_–TiO_2_ humidity sensor. Sens. Actuators B Chem..

[53] Farahani H., Wagiran R., Hamidon M.N. (2014). Humidity sensors principle, mechanism, and fabrication technologies: A comprehensive review. Sensors.

[54] Korvink J.G., Chandran L., Boltshauser T., Baltes H. (1993). Accurate 3D capacitance evaluation in integrated capacitive humidity sensors. Sens. Mater..

[55] Nellikkal M.A.N., Ahmad Z., Shakoor R.A. (2018). Organic thin-film capacitive and resistive humidity sensors: A focus review. Adv. Mater. Interfaces.

[56] Ahmadipour M., Fadzil Ain M., Ahmad Z.A. (2016). Fabrication of resistance type humidity sensor based on CaCu_3_Ti_4_O_12_ thick film. Measurement.

[57] Su P.-G., Wang C.-S. (2007). Novel flexible resistive-type humidity sensor. Sens. Actuators B Chem..

[58] Najjar R., Nematdoust S. (2016). A resistive-type humidity sensor based on polypyrrole and ZnO nanoparticles: Hybrid polymers *vis-a-vis* nanocomposites. RSC Adv..

[59] Horowitz H.H., Metzger G. (1963). A new analysis of thermogravimetric traces. Anal. Chem..

[60] Narayanan R., Laine R.M. (1998). Synthesis and characterization of precursors for group II metal aluminates. Appl. Organomet. Chem..

[61] Tahira M., Sayyad M.H., Clark J., Wahab F., Aziz F., Shahid M., Munawar M.A., Chaudry J.A. (2014). Humidity, light and temperature dependent characteristics of Au/N-BuHHPDI/Au surface type multifunctional sensor. Sens. Actuators B Chem..

