# Supplementary material for: Design and Fabrication of a Fast Response Resistive-Type Humidity Sensor Using Polypyrrole (Ppy) Polymer Thin Film Structures
Source: Polymers (Basel). 2021 Sep 7;13(18):3019. doi: 10.3390/polym13183019 (PMC8468344; doi:10.3390/polym13183019)
Supplement: Supplementary file 1 [file polymers-13-03019-s001.zip › polymers-1321120-sup.pdf]

Supplementary Information

Design and Fabrication of a Fast Response Resistive-type Humidity Sensor Using Polypyrrole (Ppy) Polymer Thin Film Structures

Mushahid Hussain [1,†], Saqib Hasnain [2,†], Nadir Ali Khan [1,†], Shehar Bano [3], Fazeelat Zuhra [4], Muhammad Ali [5], Munawar Khan [1], Naseem Abbas [6,*] and Ahsan Ali [7,*]

[1] Department of Electronics, University of Peshawar, Pakistan

[2] Department of Mechatronics Engineering, University of Engineering and Technology Taxila, Pakistan

[3] School of Mechanical Engineering, Southwest Jiaotong University, Chengdu 610031, PR China

[4] Department of Chemistry, University of Peshawar, Pakistan

[5] Department of Physics, University of Peshawar, Pakistan

[6] Sensor System Research Center, Korea Institute of Science and Technology (KIST), Seoul 136-791, Korea

[7] Department of Mechanical Engineering, Gachon University, Seongnam-Si 13120, Republic of Korea

* Correspondence: nabbas5@kist.re.kr (N.A.); ahsanali@gachon.ac.kr (A.A.)

[†] These authors contributed equally to this work

List of Figures

Figure S1 Polypyrrole polymerization scheme (a): Generation of free cation radical, (b): polymer chain propagation, (c): formation of a polymer chain.

Figure S2 Types of bonds in natural state in the benzenoid structure of Polypyrrole (a): aromatic, (b): quinonoid structure.

Figure S3 Correlation among dew/frost point, PPMv, and % RH.

Figure S4 FTIR spectra of Ppy composite

Figure S5 Real-time humidity values of the sensor

S1 Polypyrrole Polymerization Process

Pyrrole (C_4H_4NH) can easily be polymerized in to Polypyrrole ($H(C_4H_2NH)_nH$) polymer by electrochemical synthesis. First step involves the oxidation of Pyrrole and produces a free cation radical. Latter on the entire chemical process repeats itself several times and finally yields Polypyrrole. Figure S1a–c is showing the entire polymerization process of Polypyrrole. Figure S1a,b is showing two types of famous bonding structure in natural state in the benzenoid structure of Polypyrrole polymer.

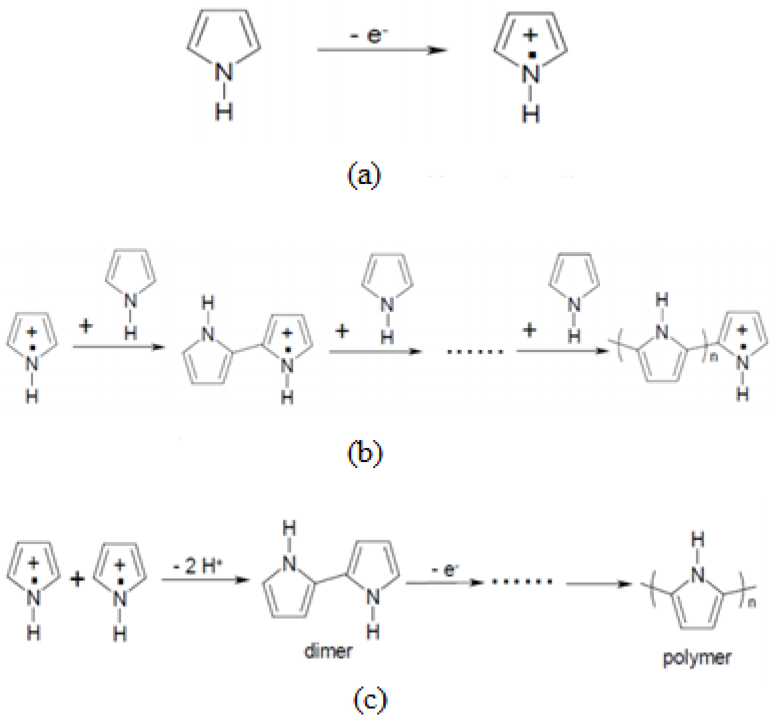

Figure S1 Polypyrrole polymerization scheme, (a): generation of free cation radical, (b): polymer chain propagation, (c): formation of a polymer chain.

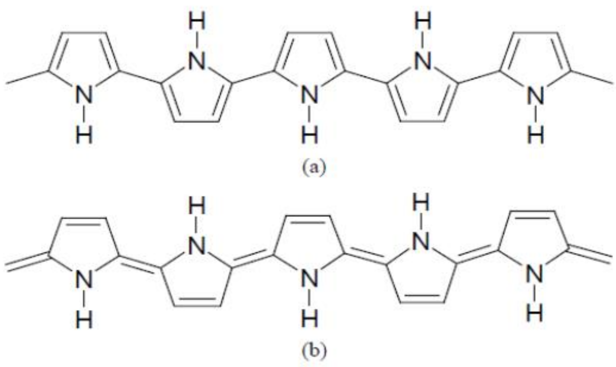

Figure S2 Types of bonds in Polypyrrole polymers (a): aromatic, (b); quinonoid structure.

S2 Humidity Types and Units

Humidity can be quantified in form of following measuring parameters:

S2.1 Relative Humidity (RH)

Relative humidity is the ratio of an actual partial pressure (P_V) to saturated pressure (P_S) at present temperature, and can be written as,

$$\% \text{ RH} = \frac{P_V}{P_S} \text{ x } 100 \quad (1)$$

It is normally expressed in percentage, and a higher percentage value means that the measuring mixture or environment is more humid. Also, RH is an important metric used in several applications like weather forecasting, dew, and fog predictions etc.

S2.2 Absolute Humidity (AH)

Absolute humidity (vapor density) is defined as; it indicates the mass of water vapors present in 1 m^3 of dry air and can be expressed as,

$$\text{AH} = \frac{m_w}{v} \quad (2)$$

AH is an absolute humidity (measured in grains/ft^3 or g/m^3) metric, where m_w is the mass of water vapors (in grams or grains) and v denotes the volume of air (in form of $ft^3 or\ m^3$).

Other less commonly used humidity measuring parameters and units are dew/frost point (D/F PT), and parts per million (PPM). Figure S3 shows a correlation in form of a scale between relative humidity, parts per million by volume, and dew/frost point. Relative humidity is a commonly used metric and it covers a higher humidity range while PPM_V and dew/frost point presents lower humidity ranges. In conclusion, relative humidity is widely used for tracing moisture measurement, whereas PPM_V , and dew/frost point helps in measuring the absolute amount of water vapors in air or gas.

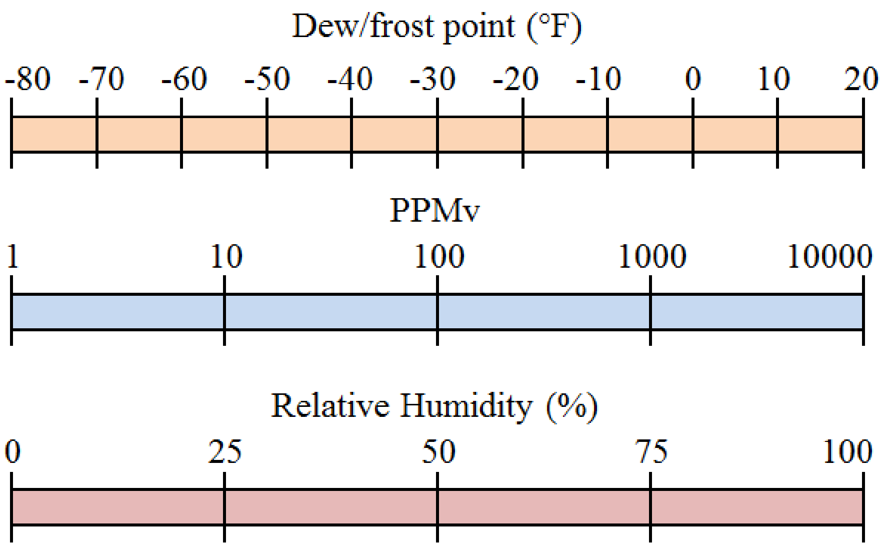

Figure S3 Correlation among dew/frost point, PPMv, and % RH.

S3 Procedure for Filtration of Synthesized Polypyrrole Composite

At first, distilled water of 100 mL was taken in to a glass beaker. After wards, synthesized Polypyrrole final composite obtained from above procedure was dropped in this distilled water and sonication process was performed for nearly 30 minutes. Then Polypyrrole final composite was taken out from sonicator and the filtration process was started as described in the following sub-sections.

S3.1 Filtration

The suspension was then passed from a filter paper. A washed and purified Polypyrrole was collected from the surface of the filter paper and was stored in a plastic bag.

S3.2 Method of Collection of Smallest Particles of Polypyrrole from Distilled Water

Smallest particles of Polypyrrole composite were collected in such a way that the distilled water was passed from a 0.22 mm syringe filter. The syringe filter passed the distilled water through it and collected Ppy particles and stored them in its body until the entire (100 mL) distilled water was passed through the filter.

S3.3 Drying of Polypyrrole Composite

Polypyrrole composite was kept in a vacuum oven for a time period of three to four hours for drying purposes, and the temperature of the vacuum oven was raised from 70 °C to 80 °C. Then, the Polypyrrole composite was poured out from the vacuum oven; this gives the Polypyrrole composite which is quite suitable for fabrication of thin film structure. Note that

for the preparation of thin films, polyvinyl alcohol (PVA) solution is also necessary which will be obtained in the next step.

S3.4 PVA Solution

Two grams of PVA ($[CH_2CH(OH)]_n$) and 20 mL distilled water was taken in a glass beaker. They were mixed, and stirring was performed. The suspension of PVA and distilled water were kept in a hot plate. Stirring was again done on it at a temperature of 140 °C, and the rotation speed was fixed at 340 RPMs. The duration of stirring was nearly 10 to 15 minutes, after that stirring process was stopped and the PVA solution was obtained, which is now suitable for sticking Polypyrrole composite on an IDE to fabricate a thin film on it.

S4 FTIR Spectra

FTIR spectrum is presenting the material group types, concentrations, and types of bonding. FTIR spectra obtained (from paragon 1000, Perkin-Elmer, USA) for this work is with a range of wave number from 4000 to 650 cm^{-1} for duration of 64 scans, and with a 2 cm^{-1} of resolution. Ppy composite sample was annealed at three different temperatures like 100 °C, 200 °C, and 300 °C, respectively. Figure 4S shows FTIR spectra of Ppy composite. The FTIR spectrum of Ppy composite has shown very strong and broad peaks at 3299 cm^{-1} given by the hydroxyl (OH) group, and at 2940 cm^{-1} given by CH_2 showing asymmetrical stretching. Furthermore, the peak observed at 1091 cm^{-1} was due to the presence of the Pyrrole group, while a narrow peak at 1734 cm^{-1} indicates the carbonyl (C=O) group stretching bond, respectively. Furthermore, the sharp and narrow intensities of peaks of Ppy composite powder confirm that the newly prepared material is of high quality and with good crystallinity.

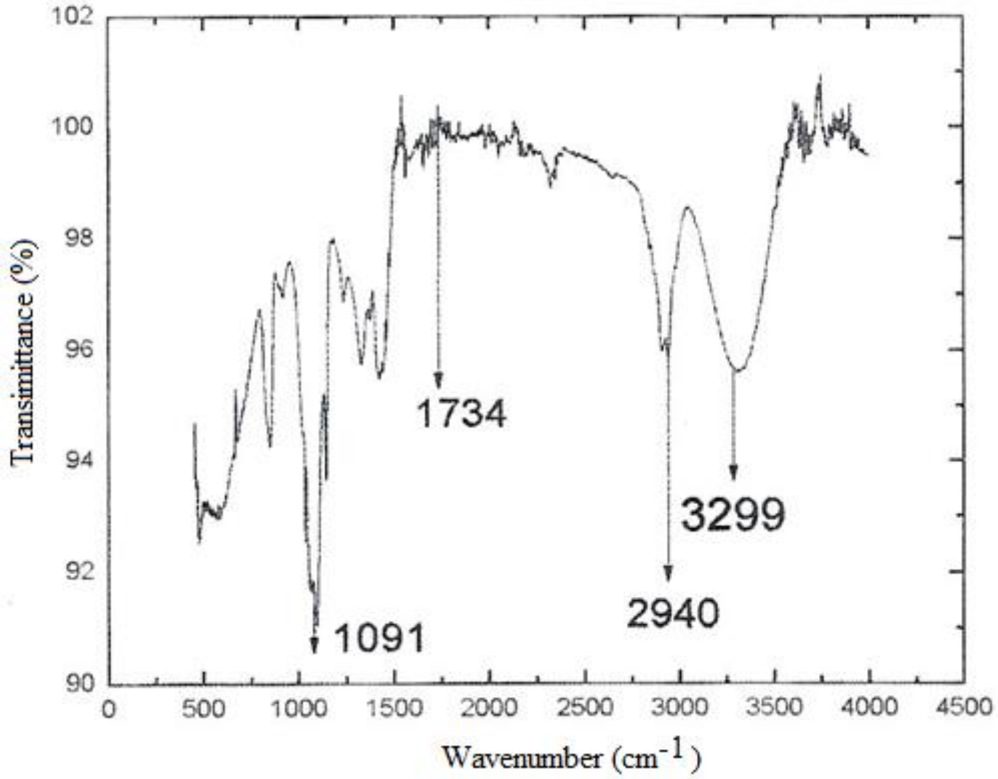

Figure S4 FTIR spectra of PPy composite.

S5 Real-time Humidity Response Values

Figure S5 shows the real-time humidity values of the sensor taken for a timespan of two days. The proper functioning of this sensor can also be verified and visualized from both the curves responses. Additionally, both the curves show a smoother response that this newly fabricated sensor is giving and showing a best performance at room temperature.

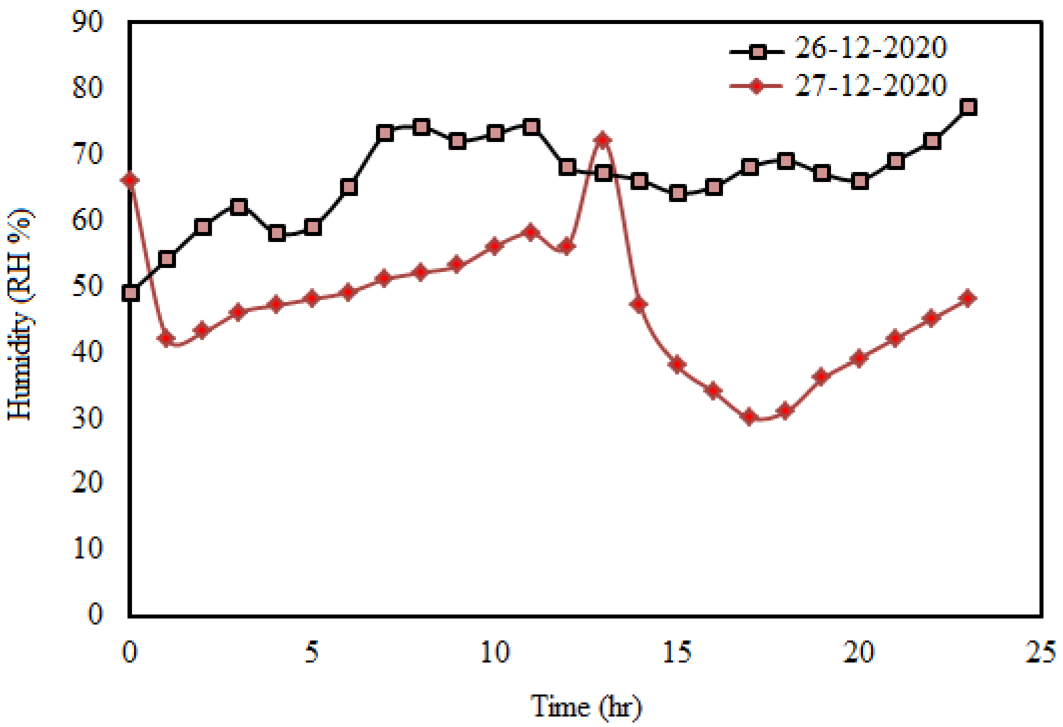

Figure S5 Real-time humidity values of the sensor.
